# Left-right compatibility in the processing of trading verbs

**DOI:** 10.3389/fnbeh.2014.00016

**Published:** 2014-01-28

**Authors:** Carmelo M. Vicario, Raffaella I. Rumiati

**Affiliations:** ^1^School of Psychology, The University of QueenslandBrisbane, QLD, Australia; ^2^Cognitive Neuroscience Sector, SISSATrieste, Italy; ^3^School of PsychologyBangor University, Bangor, UK

**Keywords:** language, economics, SNARC effect, mental accounting theory, spatial valence hypothesis

## Abstract

The research investigating the nature of cognitive processes involved in the representation of economical outcomes is growing. Within this research, the mental accounting model proposes that individuals may well use cognitive operations to organize, evaluate, and keep track of their financial activities (Thaler, 1999). Here we wanted to test this hypothesis by asking to a group of participants to detect a syntax mistake of verbs indicating incoming and going out activities related to economical profit (trading verbs), swapping (swapping verbs) and thinking (thinking verbs). We reported a left-right compatibility for trading verbs (i.e., participants were faster with their right hand while detecting verb referring to a monetary gain with respect to a monetary loss; and faster with their left hand while detecting a monetary loss with respect to a monetary gain). However, this pattern of result was not reported while detecting swapping verbs. Results are discussed taking into account the mental accounting theory as well as to the spatial mapping of valence hypothesis.

## Introduction

The interest in the nature of cognitive processes involved in the representation of economical outcomes has been growing in recent years (see e.g., Wu et al., 2012 for a recent review). Several studies in cognitive sciences and financial economics propose the inextricable interdependence between rationality and emotion (Grossberg and Gutowski, 1987; Damasio, 1994; Elster, 1998; Loewenstein, 2000; Harvey et al., 2010) in influencing human economical choices and behaviors (see also Glimcher and Rustichini, 2004). For instance, Loewenstein (2000) highlights the impact of immediate emotions, as well as wide range of visceral factors associated with them, in determining systematic behaviors that could also be amenable in a formal model. Moreover, a crucial role is played by the activation of reward-related brain areas, such as the striatum (Fehr and Camerer, 2007).

Nevertheless, other factors, beyond those mentioned above, might play a role in processing and representing economic outcomes. The mental accounting theory (Thaler, 1980), a model developed in the field of behavioral economy, proposes some intriguing suggestions in this direction. This model attempts to describe the process whereby people code, categorize and evaluate economic outcomes. According to this model, individuals use cognitive operations to organize, evaluate, and keep track of their financial activities (Thaler, 1999). Since this model holds that accounting operations are engaged in evaluating economical outcomes, one could expect a specific role of the mathematical brain processes in representing financial meanings.

A way to test this hypothesis is provided by the study of language. In fact, one could argue that the same cognitive processes active while manipulating quantity might be involved in processing financial words. Thus, following this suggestion, linguistic items such as verbs referring to monetary *gain* and/or *loss* could be conceptualized in terms of mental shifts toward higher or lower quantities or as two mental accounting operations such as *addition* and *subtraction*. This proposal originates from the evidence that Western populations are endowed with a left-to-right Mental Number Line (MNL) for representing quantities, from lower to higher, respectively (Dehaene et al., 1990). Moreover, Knops et al. (2009) have shown that during no-symbolic addition, subjects preferentially selected numbers at the upper right location, whereas during no-symbolic subtraction, they were biased toward the upper left location.

In consideration of these findings one could expect a similar left-to-right spatial encoding for the representation of linguistic terms which refer to monetary gain and loss. Accordingly, one can hypothesize that the same cognitive mechanisms underlying the representation of quantity are covertly engaged when people read verbs associable to a monetary gain or loss. Given the direct relation, in the cognitive system, between quantity (low vs. high), arithmetic operations (addition vs. subtraction) and spatial coordinates (left vs. right), one could expect to detect faster reaction times (RTs) in using the right hand while processing verbs related to a monetary *gain* with respect to verbs related to a monetary *loss* (namely trading verbs). On the other hand, one could expect faster RTs in using the left hand while processing verbs associated to a monetary *loss* with respect to verbs associated to a monetary *gain*. Participants were also performed a second block of stimuli (namely swapping verbs) which refer to verbs describing incoming and coming out outcomes perceived as an exchange. In fact, the main difference between trading and the swapping verbs is that only trading verbs explicitly suggest the meaning of “economical profit”, although a monetary outcome can be associated to both categories (e.g., money loss vs. money donation). We use of swapping verbs to create an incoming vs. going out condition in absence of high economical relevance (compared to the trading category). In this way, we could have more elements to understand whether the origin of the hypothesized left-right encoding is linked to the economical relevance of the linguistic term rather than to the incoming vs. going out meaning covertly suggested by all these verbs.

## Materials and methods

### Participants

Twenty-two right-handed graduate students (10 men, 12 women, mean age: 26 ± 7.03 years) recruited from the University of Trieste, participated in the studies after providing verbal informed consent. The experiment was performed in accordance with the ethical standards laid down in the 1964 Declaration of Helsinki. Participants received a payment of 10 Euros for having taken part in this study.

### Procedure and instruments

Using their left and right index fingers, participants were required to establish, as soon as possible and in two consecutive sessions (counterbalanced design), whether 108 verbal stimuli contained (or not) a syntax mistake. The task was identical for both trading and swapping verbs.

#### Trading verbs block

Fifty-four of 108 items were spelt correctly; of these, 18 (6 verbs × 3 trials) indicated a monetary gain and 18 (6 verbs × 3 trials) indicated a monetary loss. Moreover this block included 18 *thinking* verbs (6 verbs × 3 trials) as control items (see Table 1 for the complete list).

**Table 1:** **This table reports the complete list of items used for the three verb categories**.

							
**Trading**	**Gain verbs**	Incassare (To cash)	Riscuotere (To cash)	Ricavare (To derive)	Guadagnare (To gain)	Intascare (To rake in)	Arricchire (To enrich)
	**Loss verbs**	Pagare (To pay)	Risarcire (To compensate)	Indennizzare (To indemnify)	Perdere (To lose)	Saldare (To pay)	Impoverire (To impoverish)
**Swapping**	**Receiving verbs**	Ricevere (to receive)	Ereditare (to inherit)	Accettare (to accept)	Accoglier (to welcome)	Rilevare (to take)	Acquisire (to acquire)
	**Giving verbs**	Regalare (to give)	Donare (to donate)	Offrire (to offer)	Devolvere (to devolute)	Consegnare (to deliver)	Porgere (to hand)
**Thinking Verbs**		Ritenere (to consider)	Credere (to believe)	Supporre (to suppose)	Pensare (to think)	Immaginare (to imagine)	Sperare (to hope)

*The corsive items refer to the verbs (written in Italian) originally used in the task. In the parenthesis it is proposed the English translation*.

#### Swapping verbs block

Fifty-four of 108 items were spelt correctly; of these, 18 (6 verbs × 3 trials) indicated a receiving action and 18 (6 verbs × 3 trials) indicated a giving action. Even in this block were included 18 *thinking* verbs (6 verbs × 3 trials) as control items (see Table 1 for the complete list).

All verbs were presented in first person and in the simple present tense. Each trial was preceded by an alerting sentence (ready) lasting 500 ms and followed by fixation cross lasting 500 ms. The within subjects variable was the responding finger. Incorrect responses (Trading verbs: 2.82%; Swapping verbs: 3.05%) were not considered in the analysis.

### Statistical analysis

The RTs performance in detecting stimuli written correctly was analyzed using ANOVA for repeated measures, with VERB (trading vs. swapping), MANUAL RESPONSE (left vs. right) and MEANING (incoming, thinking and going out) as main factors. Trading and swapping verbs were presented in separated blocks. *Post-hoc* comparisons were performed using the Duncan *post-hoc* test. For all statistical analyses, a *p*-value of 0.05 was considered to be significant. Data analysis was performed using Statistica software, version 8.0, StatSoft, Inc., Tulsa, USA. We also performed a permutation analysis where we relabelled and shuffled verbs across conditions. This analysis was conducted to have an approximation of what could have happen if Swapping and Trading verbs were randomly assigned. In this case, the analysis was conducted by using Matlab software, R 2013 A version. The number of permutation selected for this procedure was 1.000; *p*-value of 0.05 was considered to be significant.

## Results

In order to evaluate the grade of familiarity of participants with the proposed verbs, they were asked to use a five point rating scale to have a subjective measure of their level of experience/familiarity. Therefore, the higher the reported score the higher the subjective experience/familiarity with a verb.

### Trading verbs

We detected a significant difference (*F*_(2,40)_ = 35.00, *p* < 0.001). *Post-hoc* comparisons revealed that both Incoming vs. Going out verbs significantly differed from the control category (*Thinking*: *M* = 4.753 ± 0.250 vs. *Incoming*: *M* = 3.531 ± 0.922 SD, *p* < 0.001; *Thinking*: *M* = 4.789 ± 0.234 vs. *Going out*: *M* = 3.515 ± 0.783 SD, *p* < 0.001), while no difference was observed between them (*p* = 0.100).

### Swapping verbs

We detected a significant difference (*F*_(2, 40)_ = 67.50, *p* < 0.001). *Post-hoc* comparison showed that both types of *Swapping Verbs* significantly differed from the control category (*Thinking: M* = 4.734 ± 0.260 vs. *Incoming*: *M* = 3.174 ± 0.749, *p* < 0.001; *Thinking: M* = 4.734 ± 0.260 vs.* Going out: M* = 3.795*, p* < 0.001). A significant difference was found also between swapping verbs (*p* < 0.001) showing that going out verbs were perceived as more familiar than incoming verbs.

**Figure 1:**
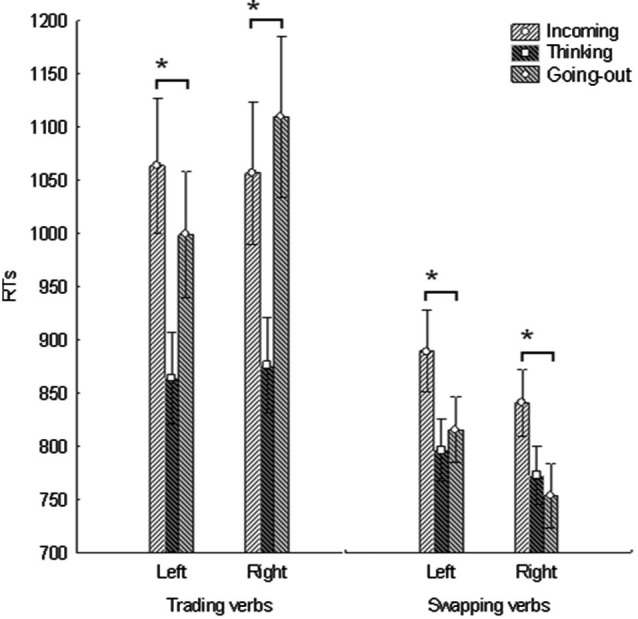
**The interplay between manual response and the comprehension of trading and swapping verbs**. Reaction times for detecting the correctly spelt items of both *trading* and *swapping* categories. Vertical bars indicate one Standard Error. The “*” indicates significant *post-hoc* comparison differences.

The analysis of RTs was conducted by excluding 2 participants from the original sample: one participant was excluded because his low performance accuracy (i.e., <80%); the other participant was excluded because he did not complete the experiment (i.e., the swapping block). According to our research hypothesis, we detected a significant result for the VERB * MEANING * MANUAL RESPONSE interaction factor (*F*_(2,38)_ = 5.24, *p* = 0.009). *Post-hoc* comparison shows a double dissociation in RTs for verbs of the trading category. In particular, participants were significantly faster in detecting going out verbs (*M* = 998.7 ± 59.69) with respect to incoming verbs (*M* = 1063.6 ± 62.94) while using their left hand (*p* = 0.013); and incoming verbs (*M* = 1056.9 ± 67.02) with respect to going out verbs (*M* = 1109.9 ± 75.74) while using their right hand (*p* = 0.042). On the other hand, *post-hoc* comparison concerning verbs of the swapping category only showed faster RTs in detecting going out items, with respect to incoming items. This was significant for both left (*p* = 0.007) and right (*p* = 0.001) hand responses (see Figure 1 for details).

This pattern of results was confirmed by the permutation analysis in almost all cases. In particular we show significant RTs difference for the trading category by comparing going out with respect to incoming verbs for the left (*p* = 0.024) and the right (*p* = 0.005) hand; We also detected a significant RTs difference for the swapping category by comparing going out with respect to incoming verbs for the right hand (*p* < 0.001). However, this difference is not significant for the left hand (*p* = 0.1727).

Ngram viewer by Google was also used to provide an idea about the frequency of use of these verbs in the Italian language. In particular, we focused on the temporal interval between the 1998 and the 2008 (i.e., the most recent temporal range available with Google Ngram viewer).

**Figure 2:**
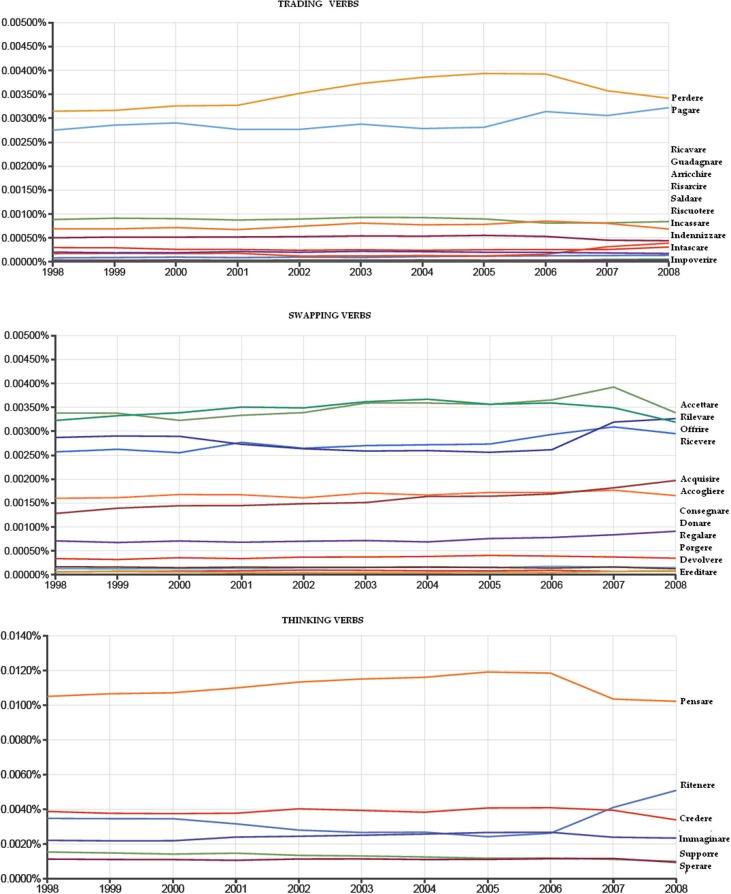
**Google Ngram viewer outputs for the three verb categories**. From top to down this figure show the output % provided by google Ngram viewer for Trading, Swapping and Thinking verbs from 1998 to 2008.

First, we performed a *t*-test analysis by comparing the Ngram viewer output (i.e., the amount of citations) provided for the 12 Trading verbs (*M* = 7921698,8) with that of the 12 Swapping verbs (*M* = 12908273,3). Results did not report a significant difference (*t* = −0.91, *p* = 0.367). We also performed two repeated measures ANOVA in which we compared the Google Ngram viewer output of Incoming, Going-out and Thinking verbs of both Trading and Swapping categories. The analysis for the Trading category documented a significant differences (*F*_(2,10)_ = 5.99, *p* = 0.01). In particular we found that Thinking verbs (*M* = 38387857,5) were more frequently cited than Incoming (*M* = 3805269,5), (*p* = 0.009) and Going out (*M* = 12038128,3) verbs (*p* = 0.030). No difference was reported by comparing them to each other (*p* = 0.448). On the other hand, we did not detect a significant difference for the swapping block, although the trend (*F*_(2,10)_ = 3,67, *p* = 0.06). Figure 2 shows a plot of the three verb categories.

## Discussion

The purpose of this study was to address the question suggested by the mental accounting theory, that is, whether people use cognitive operations for processing financial activities (Thaler, 1999). This hypothesis was addressed by studying the participants’ performance in a linguistic task.

Recently, Baroni et al. (2013) have documented a left-right compatibility by using financial words (i.e., *monopoly*, *salary*, *discount*) with faster left hand RTs while detecting words indicating loss concepts (i.e., unemployment) and, *vice versa*, faster right had RTs while detecting words indicating gain concepts (i.e., salary). However, the effect on RTs was only found when participants were required to explicitly discriminate between gain and loss, while there was failure in detecting this effect when they were required to discriminate between economic and no economic terms. On the other hand, in a further experiment, in which participants were asked to arbitrary allocate financial words along a line, the authors documented a left space preference in a spontaneous allocation of “loss” words and, *vice versa*, a right space preference in the spontaneous allocation of “gain” words. This last experiment suggests a left to right encoding for gain and loss meaning even in implicit tasks. Our study differs from the research conducted by Baroni et al. (2013) not only with respect to the adopted procedure (i.e., participants were asked to identify a syntax mistake), but also with respect to the verbal material [i.e., Baroni et al. (2013), used financial words while we used verbs which referred to incoming vs. going out outcomes, with high (i.e., trading) vs. low (i.e., swapping) economical relevance].

According to the initial prediction we found a left-to-right spatial compatibility for trading verbs. In particular, participants were significantly faster in detecting verbs indicating a monetary loss (i.e., going out) with respect to verbs indicating a monetary gain (i.e., incoming) while using their left hand; on the other hand, participants were faster in detecting verbs indicating a monetary gain with respect to verbs indicating a monetary loss while using their right hand. However, we did not detect a left-to-right spatial compatibility for swapping verbs. This suggests that the incoming vs. going out meaning implicitly associated to both verb categories might be not relevant, *per se*, in explaining the left-right compatibility found for the trading category. In fact, if this was the case, a left-right compatibility would have been detected also for the swapping category.

In the light of this argument, one could argue at least two alternative suggestions to explain the current result.

One possibility is that the left-right compatibility reported for trading verbs might reflect the higher economical relevance of this linguistic category, compared to the swapping category. As already discussed in the introduction, a dense literature (Dehaene et al., 1990; Loetscher et al., 2008; Vicario, 2012; Holmes and Lourenco, 2013; Shaki and Fischer, 2013) has repeatedly demonstrated a left-to-right mapping for low and high numbers, which is reflected in the so called SNARC effect. Accordingly, the current results can be explained assuming that when Western participants read verbs (with high economical relevance) associated to a monetary *loss*, their cognitive activation moves “leftward” as when detecting small numbers and/or performing arithmetical subtraction; *vice versa*, reading verbs (with high economical relevance) associated to a monetary *gain* activates a mental rightward shift as when detecting high numbers and while performing arithmetic addition (Knops et al., 2009). According to this interpretation our data can be intended as a support to the mental accounting theory (Thaler, 1999) stating that people use cognitive operations for processing financial activities. In fact, the current result suggests that linguistic terms referring to economics are spatially mapped similarly to numbers. This implies the suggestion that the left-right compatibility reported for trading verbs might reflect a SNARC-like effect for this linguistic material as well as for magnitude processing (Vicario and Martino, 2010).

Several arguments can be provided in support of this hypothesis. First, a common Intraparietal Sulcus (IPS) activation has been observed when participants performed calculation, linguistic and saccadic movement tasks (Sereno et al., 2001). In fact, this area has been identified by these authors as the neural correlate of the mental accounting and linguistic competence interplay; Second, learning difficulties in mathematics (i.e., developmental dyscalculia) frequently co-occur with impairments in reading (i.e., developmental dyslexia). This co-morbidity could be related to the malfunctioning of the left angular gyrus, a brain area that has been found to be affected in patients with Gerstmann (1940) who show not only acalculia but also left–right disorientation; Third, patients with cortico-basal degeneration (CBD) can show a severe difficulty in understanding small numbers as well as quantifier terms (McMillan et al., 2006). They also provided a further support to this view by performing a neuroimaging study investigating quantifier comprehension in healthy adults (McMillan et al., 2005). Semantic theorists (e.g., Szymanik and Zajenkowski, 2010) make a general distinction between first-order quantifiers, which identify a number state (e.g., “some” or “at least 3”) and higher-order quantifiers, which are those not expressible in first-order logic (e.g., “most” or “every other”). McMillan et al. (2005) reported that first-order and higher-order quantifiers both recruit right inferior parietal cortex, suggesting that a number processing component contributes to quantifier comprehension. In fact, parietal activation was also widely reported in subjects asked to perform a simple number processing (Cohen et al., 2000; Kazui et al., 2000; Pinel et al., 2001; Simon et al., 2002) or arithmetic task (Menon et al., 2000; Knops et al., 2009; Krueger et al., 2011).

An alternative, no less important, interpretation to the current results might refer to the *body-specificity hypothesis* (Casasanto, 2009, 2011; Kominsky and Casasanto, 2013; Kong, 2013), stating that people conceptualize bad and good in terms of left-right spatial encoding, according to their handedness. For example, Casasanto (2009) showed that right-handers tend to associate rightward space with positive ideas and leftward space with negative ideas (this pattern was reversed in left-handers). In fact, while trading verbs might be conceptualized as endowed of an emotionally positive (i.e., incoming) and negative (i.e., going out) meanings, all swapping verbs might be interpreted positively (e.g., “donation” is easily interpreted as “positive”, although it indicates a going out outcome).

The results reported for the swapping category provide some support to the Space-valence hypothesis. In fact, the going out verbs of the swapping category such as “to donate” (which were the most positive) show the largest advantage for the right hand, and the moderately positive incoming verbs like “to receive” show an advantage in the same direction. Moreover, the permutation test did not confirm a significant difference comparing going out with respect to incoming swapping verbs when using the left hand. This might be explained with the fact that all our participants were right handed. In fact, the spatial mapping of valence hypothesis predicts a performance advantage with the dominant hand. Therefore, according to this view, one could argue that the left-right compatibility reported in our study might be ruled by the “value” (i.e., positive vs. negative) of the presented verbs. The spatial mapping of valence hypothesis might represent a valid interpretation for explaining the current results. However, this study does not provide definitive evidence in support of this interpretation since we did not test left-handed people.

Worthy of some discussion is the difference in the familiarity ranking score provided by our participants for the three verb categories. In fact, going out swapping verbs were perceived as more familiar than incoming swapping verbs. This difference in familiarity scores for the swapping category might have played some role in the detection of these verbs, although we don’t believe that this factor might explain the absence of a left/right compatibility for this linguistic category.

Our study bears some important limitations that might be addressed in future works. First, we did not collect any ranking about the economical relevance subjectively associated to the verbs presented in this research. Second, trading and swapping verbs were administered in separated blocks. This might represent an issue since verbs within blocks might have interacted such as cueing. In fact, thinking verbs, which were used as a control condition, were detected faster in the swapping block than in the trading block. However, the permutation analysis suggests that the reported effects for the trading category are not related to the blocked design. Finally, we did not test RTs performance in left handed participants, this because our research goal was testing the existence of a SNARC like-effect for linguistic items associated to the economical profit category (i.e., trading verbs).

Further investigations including brain imaging and non-invasive brain stimulation methods, but also left-handed participants are needed to clarify whether the current results underlie the linguistic representation of economical outcomes.

## Conflict of interest statement

The authors declare that the research was conducted in the absence of any commercial or financial relationships that could be construed as a potential conflict of interest.
